# Supplementary material for: GOLM1 and FAM49B: Potential Biomarkers in HNSCC Based on Bioinformatics and Immunohistochemical Analysis
Source: Int J Mol Sci. 2022 Dec 6;23(23):15433. doi: 10.3390/ijms232315433 (PMC9737887; doi:10.3390/ijms232315433)
Supplement: Supplementary file 1 [file ijms-23-15433-s001.zip › ijms-1978048-supplementary.pdf]

Table S1. Clinicopathological characters of HNSCC in TMAs

Characteristics	Sub-characteristics	N (%)
Age(years)		64(30-82)
Gender	Male	75(0.4)
	Female	8(9.6)
Tumor size(T)	T1	12(14.5)
	T2	35(42.2)
	T3	29(34.9)
	T4	7(8.4)
Lymph node metastasis(N)	N0	44(53.0)
	N1	28(33.7)
	N2	11(13.3)
Distant metastasis(M)	M0	83(100.0)
	M1	0(0.0)
Grade	I	32(38.6)
	II	27(32.5)
	III	24(28.9)
TNM stage		
	I+II	26(31.3)
	III	41(49.4)
	IVA	16(19.3)
The location	throat	33(39.7)
	head and neck	13(15.7)
	tongue	16(19.3)
	mouth	16(19.3)
	the others	5(6.0)
Total		83(100.0)

Table S2. Associations of GOLM1 and FAM49B expression with clinicopathological parameters of HNSCC tissues

Parameters	Cases (n=81)	GOLM1 in tumour cells			FAM49B in tumour cells		
		Positive	Negative	P value	Positive	Negative	P value
Gender				0.784*			0.826*
Male	73	54	19		11	62	
Female	8	5	3		2	6	
Age				0.117*			1.000*
<=64	63	49	14		10	53	
>64	18	10	8		3	15	
Depth of invasion(T)				0.163			0.457
T1,T2	45	30	15		6	39	
T3,T4	36	29	7		7	29	
Lymph node metastasis(N)				0.482			0.446
N0	42	32	10		8	34	
N1,N2	39	27	12		5	34	
Grade				**0.004**			0.898*
I,II	58	37	21		10	48	
III	23	22	1		3	20	
TNM stage				0.596			0.277
I+II	24	16	8		5	19	
III	41	30	11		4	37	
IVA	16	13	3		4	12	

*: Continuity correction P value

Table S3. Combined score of the relationship between GOLM1 and its predicted functional partners.

Partners of GOLM1	Coexpression	Coexpression (Other organisms)	Experiments	Textmining	Textmining (Other organisms)	Combined score
CLU	0.061			0.914	0.041	0.915
GOLPH3				0.820		0.820
EGFR	0.088		0.270	0.714		0.793
NAA35				0.418	0.621	0.769
AFP				0.736		0.736
ENDOD1				0.143	0.642	0.680
KATNAL1				0.166	0.625	0.673
GOLIM4				0.669		0.669
AMACR				0.565	0.256	0.662
GPC3				0.652		0.652

Table S4. Combined score of the relationship between FAM49B and its predicted functional partners.

Partners of FAM49B	Coexpression	Coexpression (Other organisms)	Experiments	Textmining	Textmining (Other organisms)	Combined Score
ACTR3	0.673	0.063				0.680
MTAP			0.632			0.632
LRRC40				0.341	0.463	0.630
ANGPTL7				0.181	0.563	0.626
ANKS1A	0.073		0.593			0.606
ANKS1B	0.073		0.593			0.606
TINAG		0.067		0.276	0.382	0.546
TEX2				0.270	0.403	0.545
CLDN12				0.155	0.483	0.544
PTGES3	0.525					0.525

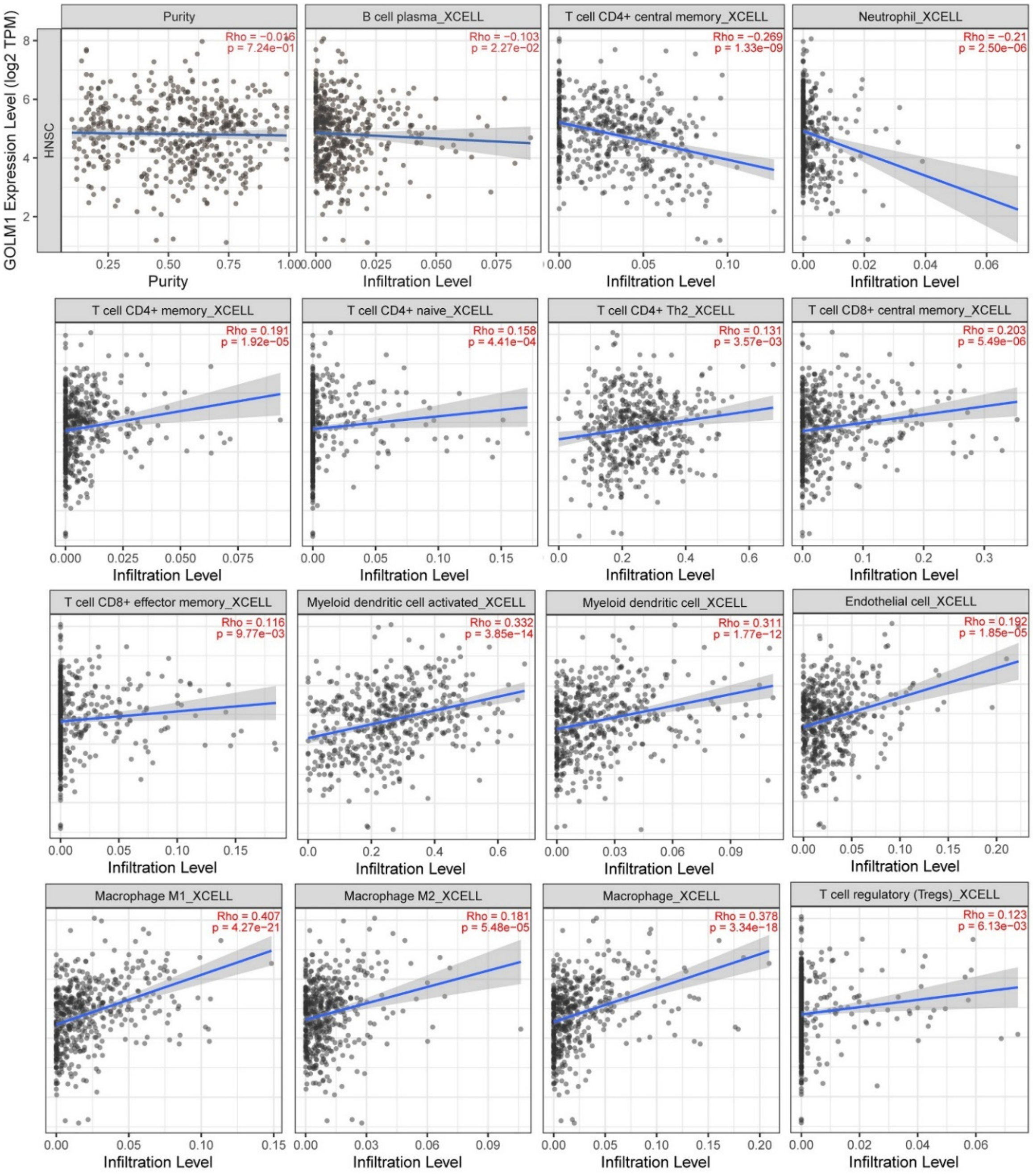

Figure S1. Correlation of GOLM1 expression with tumor purity and with the infiltration level of different immune cells estimated by TIMER2.0 in HNSCC. GOLM1 expression was positively correlated to T cell CD8$^+$ effector memory, T cell CD8$^+$ central memory, T cell CD4$^+$ naive, T cell CD4$^+$ memory, T cell CD4$^+$ Th2, regulatory T cells (Tregs), endothelial cell, macrophage, macrophage (M1, M2), myeloid dendritic cell, and myeloid dendritic cell activated. GOLM1 expression was negatively correlated with T cell CD4$^+$ central memory, B cell plasma, Neutrophil. The partial Spearman's correlation and statistical significance are shown on the top right corners.

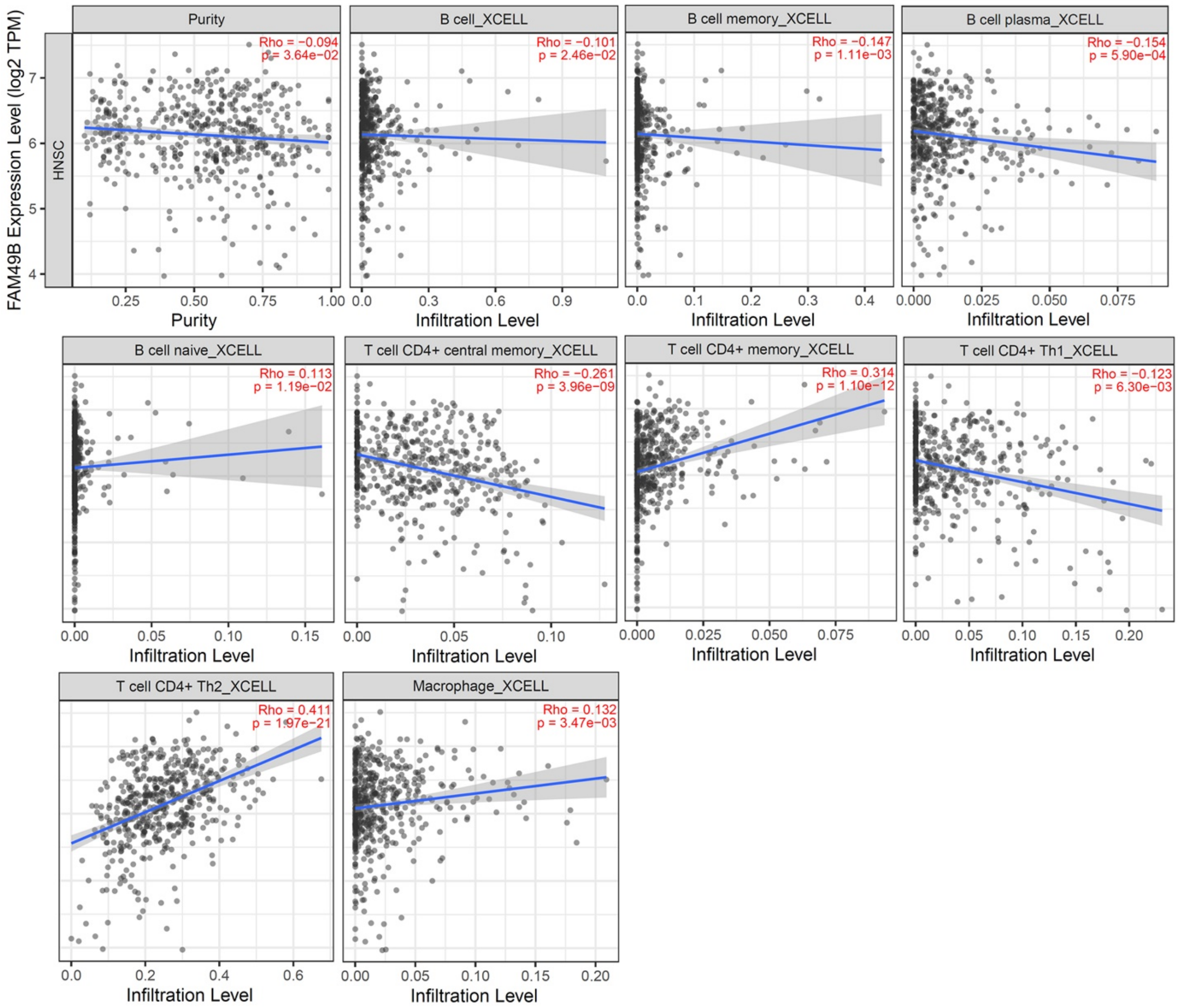

Figure S2. Correlation of FAM49B expression with different immune cells estimated by TIMER2.0 in HNSCC. FAM49B expression was positively correlated to T cell CD4^{+} memory, T cell CD4^{+} Th2, B cell naïve and Macrophage, while it was negatively related to T cell CD4^{+} central memory, T cell CD4^{+} Th1, B cell, B cell memory and B cell plasma. The purity-corrected partial Spearman correlation and statistical significance are shown on the top right corners.

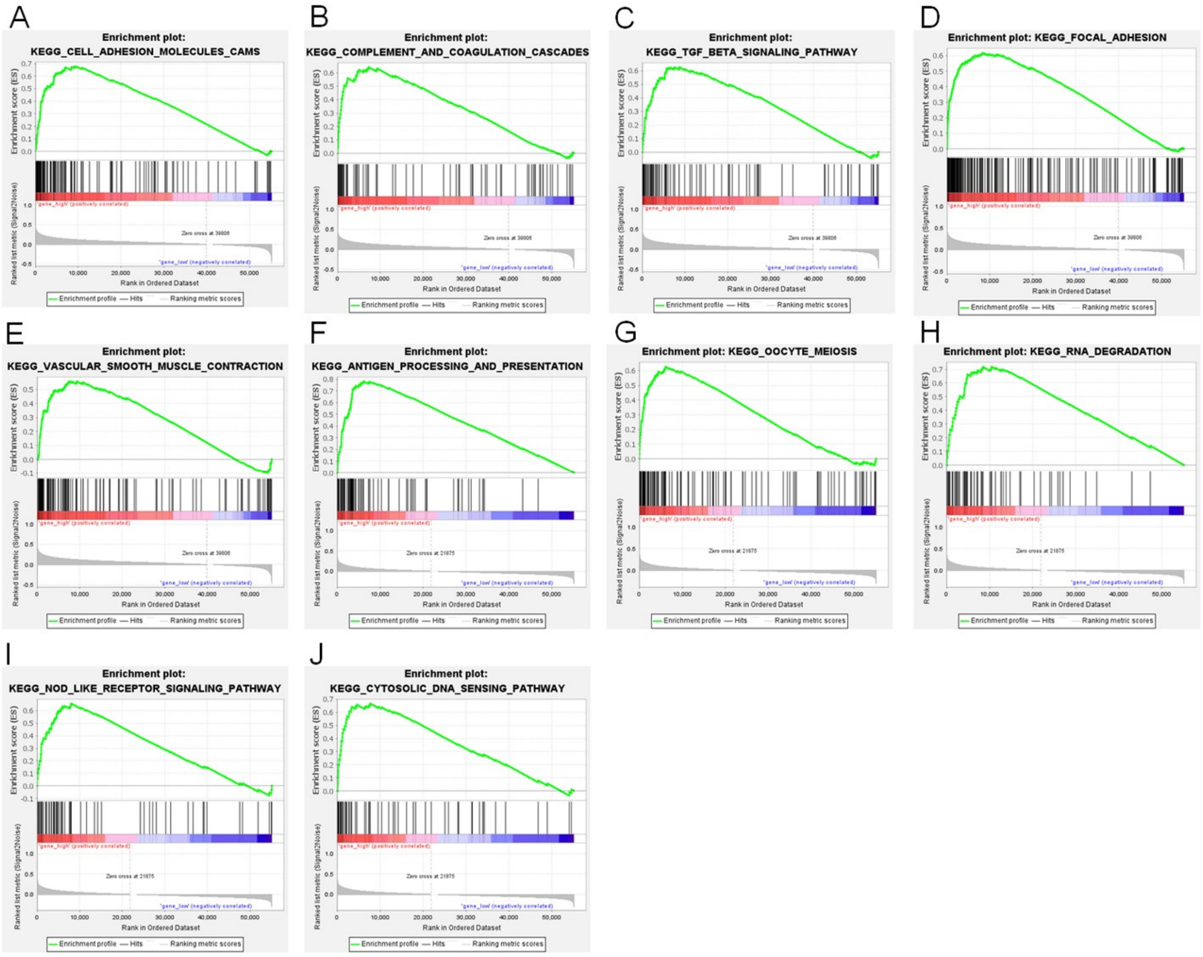

Figure S3. Top 5 pathways of GOLM1 and FAM49B genes. (A-E for GOLM1) Gene set enrichment plots of (A) cell adhesion molecules (CAMs), (B) complement and coagulation cascades, (C) TGF-beta signaling pathway, (D) focal adhesion, and (E) vascular smooth muscle contraction in HNSCC cases with high GOLM1 expression. (F-J for FAM49B) Gene set enrichment plots of (F) antigen processing and presentation, (G) oocyte meiosis, (H) RNA degradation, (I) NOD-like receptor signaling pathway, and (J) cytosolic DNA-sensing pathway in HNSCC cases with high FAM49B expression.
